# Mechanisms Underlying Rare Inherited Pediatric Retinal Vascular Diseases: FEVR, Norrie Disease, Persistent Fetal Vascular Syndrome

**DOI:** 10.3390/cells12212579

**Published:** 2023-11-05

**Authors:** Vincent Le, Gabrielle Abdelmessih, Wendy A. Dailey, Cecille Pinnock, Victoria Jobczyk, Revati Rashingkar, Kimberly A. Drenser, Kenneth P. Mitton

**Affiliations:** 1 Eye Research Institute, Oakland University, Rochester, MI 48309, USA; 2 Oakland University William Beaumont School of Medicine, Rochester, MI 48309, USA; 3 Associated Retinal Consultants P.C., Royal Oak, MI 48073, USA

**Keywords:** FEVR, Norrie disease, persistent fetal vascular syndrome, norrin, *NDP*, *FZD4*, *LRP5*, *TSPAN12*, *ZNF408*, *KIF11*, *CTNND1*, *CTNNA1*, *ECM1*, retinal endothelial cell, retinal vasculature, blood-brain-barrier, genetic disease mechanisms

## Abstract

Familial Exudative Vitreoretinopathy (FEVR), Norrie disease, and persistent fetal vascular syndrome (PFVS) are extremely rare retinopathies that are clinically distinct but are unified by abnormal retinal endothelial cell function, and subsequent irregular retinal vascular development and/or aberrant inner blood-retinal-barrier (iBRB) function. The early angiogenesis of the retina and its iBRB is a delicate process that is mediated by the canonical Norrin Wnt-signaling pathway in retinal endothelial cells. Pathogenic variants in genes that play key roles within this pathway, such as *NDP, FZD4, TSPAN12,* and *LRP5,* have been associated with the incidence of these retinal diseases. Recent efforts to further elucidate the etiology of these conditions have not only highlighted their multigenic nature but have also resulted in the discovery of pathological variants in additional genes such as *CTNNB1, KIF11,* and *ZNF408,* some of which operate outside of the Norrin Wnt-signaling pathway. Recent discoveries of FEVR-linked variants in two other Catenin genes (*CTNND1*, *CTNNA1*) and the Endoplasmic Reticulum Membrane Complex Subunit-1 gene (*EMC1*) suggest that we will continue to find additional genes that impact the neural retinal vasculature, especially in multi-syndromic conditions. The goal of this review is to briefly highlight the current understanding of the roles of their encoded proteins in retinal endothelial cells to understand the essential functional mechanisms that can be altered to cause these very rare pediatric retinal vascular diseases.

## 1. Introduction

**Figure 1:**
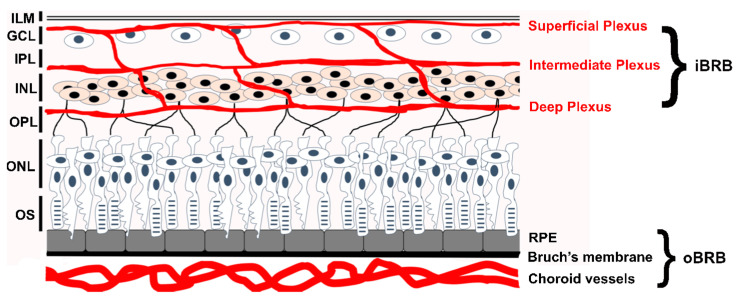
Location of the inner-Blood-Retinal-Barrier (iBRB) and retinal endothelial cells. The neural retina comprises several distinct layers. In the orientation of this diagram, light enters from the top and passes through the retinal layers to reach rod and cone opsins in the outer segments (OS) of photoreceptors. Photoreceptor nuclei form the Outer Nuclear Layer (ONL). Photoreceptor cells form synaptic connections with downstream inter-neurons in the Outer Plexiform Layer (OPL). Nuclei of Bipolar Cells, Horizontal Cells, and some Amacrine Cells comprise the Inner Nuclear Layer (INL). In the Inner Plexiform Layer, the Bipolar and Amacrine Cells form synaptic contacts with Ganglion Cells in the Ganglion Cell Layer (GCL). An Inner-Limiting Membrane (ILM) lays on top of the retina and the superficial plexus. Two different blood supplies support the neural retina and both have a high-barrier character, which are collectively the Blood-Retinal-Barrier (BRB). The outer-BRB (oBRB) sustains photoreceptor cells and Retinal Pigment Epithelial (RPE) cells and is formed by the fenestrated choroidal vasculature, Bruch’s membrane and the RPE. The RPE cells, not the choroidal endothelial cells, provide the high-barrier nature of the outer-BRB. The inner-BRB (iBRB) sustains neurons of the inner retina and comprises three microvascular beds, the superficial, intermediate, and deep plexus (shown by red tracts). These are collectively referred to as the neural retinal vasculature. The endothelial cells of the neural retinal vasculature are responsible for the high-barrier nature of the iBRB.

The retinal endothelial cell (REC) plays a central role in the developmental formation of the mammalian neural-retinal vasculature [1]. A retinal specific growth factor, Norrin, is essential for stimulating the proliferation of the retinal vasculature and recruitment of mural cells [2]. Genetic variants that change normal REC function can potentially impact the development of the entire neural retina and its function. At maturity, three interconnected microvascular beds support the inner neural retina: the Superficial Plexus in the Ganglion Cell Layer (GCL)/Nerve Fiber Layer interface, the Intermediate Plexus on the inner-side of the Inner Nuclear Layer (INL), and the Deep Plexus on the outer-side of the INL. See Figure 1. We recommend the review by Selvam et al. (2017) for those interested in the topic of retinal vascular development [3].

Inner-retinal neurons (Bipolar Cells, Horizontal Cells, Amacrine Cells, and Ganglion Cells) are fully dependent on this inner-retinal vasculature for gas, nutrient, and waste exchange. Photoreceptors are less reliant on this vasculature and are supplied by the choroidal blood supply, which is located on the opposite side of the Retinal Pigment Epithelium (RPE). This fact is well demonstrated by the mouse Oxygen-Induced Retinopathy model where inner-retinal neurons are lost during post-natal retinal development in zones that become avascular before neovascular growth restores the blood supply. OCT and ERG analysis shows that even with severe inner retinal neuron loss, the photoreceptors are not lost in any substantial numbers, and the photoreceptors remain responsive to light [4,5,6]. This is important for this review because FEVR/Norrie disease impacts the neural retinal vasculature due to effects within retinal endothelial cells.

During normal retinal development, the superficial layer extends first from the optic nerve towards the peripheral retina, resulting from proliferation of advancing retinal endothelial cells [1]. Vertical branch sprouts form from this layer and follow guidance cues from Muller Glia Cells, which extend from the ILM to the outer side of the ONL. The deep plexus then extends horizontally ahead of the intermediate plexus, which forms last. Active proliferation of Retinal Endothelial Cells (RECs) is essential for the formation of this vasculature and, after maturation, RECs are essential components of the neurovascular unit that supports the formation of the inner blood-retinal-barrier (iBRB) and its high-barrier nature. The iBRB is a highly selective barrier like the Blood-Brain-Barrier (BBB), and indeed, the neural retina is part of the Central Nervous System (CNS).

Concurrent with maturation of the developing retina, the hyaloid vasculature regresses, which is a temporary blood supply that runs from the optic nerve head to the posterior lens capsule to feed a capillary network called the Tunica Vasculosa Lentis. This embryonic vasculature is required to sustain the developing lens, an avascular tissue, until the onset of aqueous humor production to support the lens. Human patients, especially males, without functional Norrin (X-linked) may also display delayed or failed regression of this normally embryonic blood supply in addition to failed retinal vascular development. This is a condition known as Persistent Fetal Vascular Syndrome (PFVS).

The high-barrier nature of the neural retinal endothelium is a result of specific adaptions to decrease the permeability of the endothelium both between and through the cells. A relatively high-barrier to paracellular transport, between cells, is provided by high concentrations of adherens-junctions and tight-junctions between neighboring cells [7]. Reduced concentration of plasmalemma vesicles, which are caveolae vesicles associated with Caveolin, is responsible for a lower rate of transcytosis, transport through retinal endothelial cells [8]. Unlike choroidal endothelial cells, retinal endothelial cells are not fenestrated. The iBRB can become compromised in certain pathological eye diseases such as diabetic retinopathy, which manifests clinically as increased microvascular permeability and subsequent retinal hemorrhages [9]. Increased concentration of Vascular Endothelial Growth Factor-A is a major driver of barrier loss from disruption of the adherens-junctions and tight-junctions. VEGFA was also shown to increase the concentration of caveolae in bovine RECs [10].

FEVR (Familial Exudative Vitreoretinopathy) and Norrie disease are inherited disorders that impair development of the neural retina’s vasculature. In Norrie disease and FEVR (Familial Exudative Vitreo-Retinopathy), there is partial to complete failure of retinal vasculature formation, resulting in regions of peripheral retinal tissue that remain avascular and hypoxic [11,12,13]. FEVR was first described in 1969 by Criswick and Schepens, and can result in significant blindness from neovascularization, retinal traction, retinal folding, retinal detachments, and vitreous hemorrhage [14]. FEVR may also be milder with the presence of most of the retinal vasculature, but with the loss of its normally high-barrier character and even leakiness. Persistent Fetal Vasculature Syndrome (PFVS) may also result, especially from variants impacting the gene for Norrin, where regression of the temporary hyaloid vasculature is incomplete to varying degrees [11].

These conditions, especially FEVR, can present with a broad range of severity and progression, even between siblings with the same FEVR-linked variant. Reviewing the variable phenotypic penetrance would require much clinical retinal imagery that is beyond the scope of this review. However, we can refer the reader to Ranchod et al. (2011) for a succinct review of the clinical presentation of FEVR and a description of the clinical staging system for FEVR [15]. Briefly, the severity of FEVR begins with the presence of avascular peripheral retina (stage 1). Stage 2 includes the presence of retinal neovascularization without exudate (stage 2A) or with exudate (stage 2B). Stage 3 involves extramacular detachment without exudate (stage 3A) or with exudate (stage 3B). Stage 4 includes macular retinal detachment without exudate (stage 4A) or with exudate (stage 4B). The most severe stage 5 marks total retinal detachment.

Norrie disease, Coats disease, retinopathy of prematurity (ROP), and familial exudative vitreoretinopathy (FEVR) belong to a family of rare retinopathies that are characterized by irregular vascularization or even lack of vascularization of the retina [16]. Our group has contributed to continuing efforts to identify variants in several genes that play a role in the pathogenesis of these diseases, which include *NDP*, *FZD4*, *TSPAN12*, and *LRP5*, members of the canonical Norrin Wnt-signaling pathway [17]. Recent studies have also uncovered additional genes, some that have no direct participation in the Norrin-signaling pathway. They include *CTNNB1*, which encodes the canonical Wnt-signaling transcription factor ß-catenin [18]; *KIF11*, which codes for kinesin-motor protein-11, KIF11, active during mitosis [19]; and *ZNF408*, which encodes a zinc-finger rich transcription factor (ZNF408) that has heightened expression in the developing eye [20]. More recently, two other Catenin genes (*CTNND1, CTNNA1*) and subunit-1 of the Endoplasmic Reticulum Membrane Complex (*EMC1*), previously linked to cancers and multi-syndromic disease, appear to also have variants that can result in FEVR-like phenotypes in human patients [21,22,23].

Despite their distinctively unique cellular roles, these genes all share the following characteristic: variants in their coding sequence can negatively impact their essential functions in the retinal endothelial cell, resulting in aberrations of retinal vascular development and/or maintenance of the inner-BRB. Furthermore, the degree to which they are limited to their specific impact on the neural retinal endothelium, versus multi-syndromic pathologies, tends to reflect the relative specificity of their expression in the retinal endothelium and other tissues. This review will provide a brief overview of each of these genes, including the general structure and function of the respective proteins, to better understand how their variants may impact the retinal endothelium.

## 2. Genes and Proteins

For ten genes, we have included a figure that maps the location of many known pathogenic and likely-pathogenic variants relative to known functional protein domains. The protein domain variant figures were constructed using MacVector Pro 18.6.1 (MacVector Inc., Apex, CA, USA) and Photoshop 2024 (Adobe Inc., San Jose, CA, USA) software. Please note that variants of *uncertain consequence* were not included in our figures, but the reader can also explore those in the UniProt database (https://www.uniprot.org (accessed on 27 October 2023)) or other databases with variant information [24]. Locations of pathogenic and likely pathogenic amino acid variants are displayed in our figures using the format of Bateman et al. (2023) [24]. The reader should also keep in mind that variant information is not static and regular reference to online databases is recommended in addition to searches of the recent literature. It is now possible for novel likely pathogenic variants to enter variant databases directly from genetic testing results that do not appear in the literature. Below, we review the seven earlier FEVR-linked genes, followed by three more recently linked genes in the following order: *KIF11*, *ZNF408*, *CTNNB1*, *NDP*, *FZD4*, *LRP5*, *TSPAN12, EMC1, CTNNA1,* and *CTNND1*.

### 2.1. KIF11

**Figure 2:**
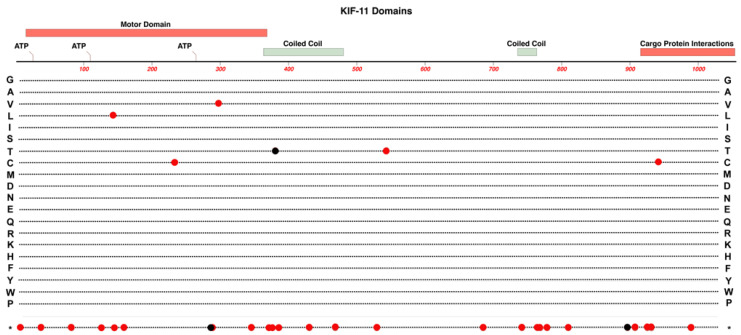
Structure and functional domain map of variants in the KIF-11 protein. Key features of the KIF-11 amino acid sequence include an ATP-binding motor-domain region, coiled-coil domains for multimer formation, and a C-terminal domain for cargo-protein interactions. Over 30 pathogenic and likely pathogenic variants are indicated by red circles with the variant amino acid indicated by the standard single-letter amino acid code. Multisyndromic with potential ocular effects (red circles) and non-ocular (black circles) disease variants are shown. The bottom row * indicates the location of nonsense (stop codon) variants. These and many other non-pathogenic variants can be explored using the UniProt database: https://www.uniprot.org/uniprotkb/P52732 (accessed on 27 October 2023).

*KIF11* is located on Chr-10 and encodes for Kinesin Family member-11 (also known as Eg5), a homo-tetrameric motor protein that is involved in the formation of spindle polarity during mitosis [25]. KIF-11 consists of three major functional components: an N-terminal motor domain, two coiled-coil domains, and a cargo protein interaction domain at the C-terminal end. A structural map of KIF-11 and the locations of most known pathogenic and likely-pathogenic protein-altering variants are shown in Figure 2.

Structurally, Kinesin Motor family proteins form dimers or tetramers with the coiled-coiled domains of each monomer entwined with the equivalent domains of their multimeric partners. This elongated structure also serves as a long connecting towbar between the motor-domain region and the cargo binding domain. As such, most of the protein’s amino acid sequence is important for movement of KIF-11 along microtubules, correct multimeric formations, and for the tethering of any required cargo. As a ubiquitously expressed protein, variants in KIF-11 are associated with multiple developmental syndromes. Heterozygous variants in this gene have been linked to microcephaly with or without chorioretinopathy, lymphoedema, or mental retardation (MCLMR), a rare autosomal dominant disease [26,27]. However, a study by Li et al. (2016) discovered an association with KIF-11 variants and the development of familial exudative vitreoretinopathy [19]. Variants included L171V, Q525*, S936*, and R1025G [19]. The data suggested that there was a trend between mutations in KIF-11 and more severe phenotypic outcomes of FEVR—all probands in one study with KIF-11 variants were diagnosed with either stage 4 or 5 FEVR.

Generally, disease-causing KIF-11 variants result in multiple syndromic conditions in addition to impacting development of the retinal vasculature. This is congruent with the fact that KIF-11 appears to be involved in the process of spindle formation and organization during cell division, a fundamental cellular process not limited to the retinal endothelial cell. It is presumed that the major impact of these disease-causing variants is mostly impacting the normal proliferation and migration of RECs in the developing neural retinal vasculature. Early post-natal, endothelial cell-specific knockout of KIF-11 in the mouse was reported to impair the formation of the retinal and cerebellar vasculature [28]. The capacity of EC cells for ß-catenin-mediated intracellular signal transduction was not impacted. Wang and co-authors have hypothesized that the cerebral and retinal microvasculature may be more sensitive because these vascular beds are the most rapidly growing later in embryonic development.

### 2.2. ZNF408

**Figure 3:**
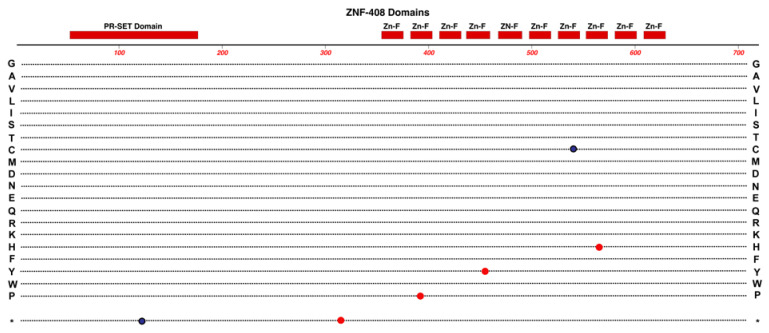
Structure and functional domain map of variants in the ZNF-408 protein. Key features include a PR-SET domain and 10 zinc-finger domains. Several pathogenic and likely pathogenic variants are indicated with the variant of amino acid indicated by the standard single-letter amino acid code. FEVR (red circles) and Retinitis Pigmentosa-72 variants (black circles) are shown. The bottom row * indicates the location of nonsense (stop codon) variants. These variants and many non-pathogenic variants can be explored in the UniProt database: https://www.uniprot.org/uniprotkb/Q9H9D4 (accessed on 27 October 2023).

*ZNF408* is located on Chr-11 and encodes a protein consisting of one PR-SET domain and 10 zinc (Zn)-finger domains. See Figure 3. This protein belongs to the PRDM family (PRDI-BF1 and RIZ homology domain-containing) of transcription factors, which are characterized by the presence of a PR domain followed by a variable number of Zn-finger repeats [29].

The PR-SET domain is involved in the recruitment of chromatin remodeling factors such as those modulating DNA-methylation. Multiple Zn-finger motifs occurring in close sequence are known to provide specific structures for binding interactions with other proteins or for binding to specific DNA-sequences. Members of this protein family can act as transcription factors, regulating a diverse range of function-related gene expression and some aspects of DNA repair [30]. Changes to the coding sequence of Zn-finger proteins have been associated with aberrations in tissue development, cancer, retinitis pigmentosa, and recent genetic studies have identified several variants associated with FEVR [20,31]. For example, the H455Y variant was identified in a family with autosomal-dominant FEVR [32].

Studies in zebrafish have confirmed that some of these variants resulted in irregular retinal vasculature development, while studies using human umbilical vascular endothelial cells (HUVECs) revealed that variants in ZNF408 reduced endothelial cell capacity for angiogenesis [20,32]. While further research is required to identify the specific gene expression changes associated with variants of ZNF408, one hypothesis suggests that variants in ZNF408 result in the decreased transcription of genes responsible for the formation of new capillaries in response to hypoxia [32].

### 2.3. CTNNB1

**Figure 4:**
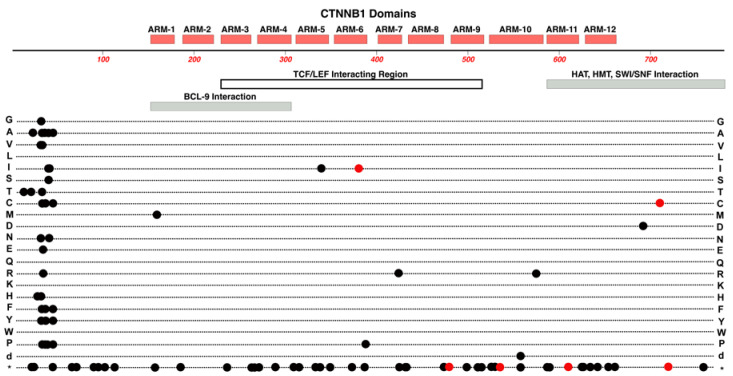
Structure and functional domain map of variants in the ß-catenin protein. Key structural features include 12 imperfect ARM repeats. The protein has numerous regulatory protein interaction partners that bind ß-catenin in separate domains but also in some overlapping regions. The N-terminus is essential for binding with alpha-Catenin as well as the beta-TrCP ubiquitin ligase that targets ß-catenin for proteosome degradation. Pathogenic and likely pathogenic variants linked to FEVR (red circles) and non-FEVR (cancer, black circles) conditions are indicated by marking the linked variant amino acid. The variant marked on row d indicates the position of a large in-frame deletion. The bottom row * indicates the location of nonsense (stop codon) variants. These and other non-pathogenic variants can be explored in the UniProt database: https://www.uniprot.org/uniprotkb/P35222 (accessed on 27 October 2023).

Located on Chr-3, *CTNNB1* encodes the protein ß-catenin. Before ß-catenin variants were reported for FEVR, many variants of this gene were already associated with cancers. The roles of ß-catenin can be relatively complex because of its regulatory roles in cell-to-cell adhesion and its pivotal role in canonical Wnt-signaling. Many cancer-related variants are clustered in the N-terminal domain of ß-catenin where they prevent the normal regulatory targeting of the protein to degradation at the proteosome. See Figure 4. This effectively results in abnormally constant hyperactivity of ß-catenin, which promotes cancer-cell proliferation [33]. In addition to its central role in Wnt-signaling, most ß-catenin is associated with E-Cadherin of the adherens-junctions in endothelial cells [34].

Most of the protein’s amino acid sequence comprises 12 ARM motifs, with each ARM region forming an alpha-helical structure. These multiple helixes are stacked together laterally, like logs, with this entire stack also twisted into a higher-order helical structure. Most of the length of this large super-structure forms a groove that provides for interactions with proteins such as TCF/LEF transcription factors [33]. For an excellent and detailed review of ß-catenin’s structures, we refer the reader to Huber and Weis (2001) [34]. While most variants in CTNNB1 are cancer-promoting and located near the N-terminus, studies have reported FEVR- and PFVS-linked variants. Two such examples are a frame-shift-causing five base-pair insertion and the A295G variant in probands with FEVR and PFVS, respectively [18,35].

### 2.4. NDP

NDP (Norrie Disease Protein, Norrin) is a cysteine-rich protein that serves as the ligand for the Norrin Wnt-signaling pathway. Norrin has been found to function as an angiogenic factor and as a neuroprotective growth factor [36]. Norrin mediates angiogenesis partly through the induction of insulin-like growth factor-1 [37]. The *NDP* gene is located on Chr-X and subsequently, symptomatic probands are more often male. While associated with Norrie disease, missense variants in NDP are also associated with diagnosed FEVR, both X-linked and sporadic [38]. Pathologic variants in NDP have been associated with several vascular retinopathies, including Norrie disease, FEVR, persistent fetal vasculature syndrome (PFVS), retinopathy of prematurity (ROP), and Coats disease [16]. While these retinopathies are largely limited to the retinal vasculature, Norrie disease exhibits the most severe manifestations with retinal dysgenesis and is unique in its association with several additional symptoms, including intellectual disability, seizures, alterations to peripheral vascular structure, and gradual hearing loss [16].

**Figure 5:**
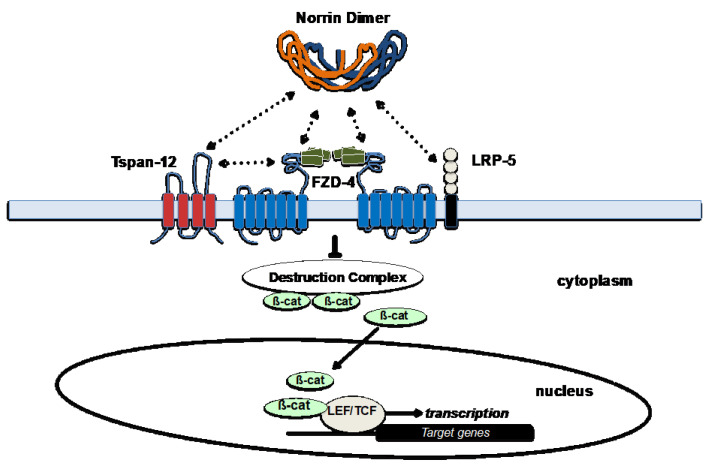
The Norrin Wnt-signaling pathway in retinal endothelial cells. The main functional proteins in the Norrin Wnt-signaling pathway, which is active in neural retinal endothelial cells. Norrin’s binding affinity for FZD-4 is increased when the co-receptors Tspan-12 and LRP-5 are present. Direct protein-protein interactions are indicated between various partners in the complex by dashed double-arrows. Specific extracellular domains and surfaces of proteins within this ligand/receptor complex provide the various interactions and the synergy which makes retinal endothelial cells uniquely sensitive to Norrin. Norrin binding blocks activity of the Destruction Complex, which reduces the delivery of ß-catenin to proteosome-degradation. The resulting increase in cytoplasmic ß-catenin and its translocation into the nucleus leads to interaction with LEF/TCF transcription factors to regulate the expression of target genes. General structural domains and features are illustrated for Norrin, FZD-4, LRP-5, Tspan-12, including transmembrane domains, extracellular loops, and other extracellular domains that are involved in protein interactions.

The Norrin protein is secreted by retinal Müller glial cells and the Norrin homodimer binds to FZD-4, which triggers canonical Wnt-signaling that governs angiogenesis in the retina and the inner ear [39,40]. TSPAN-12 and LRP-5 are essential co-receptors that enhance this binding. See Figure 5. Studies have identified the specific interacting regions between Norrin and FZD-4 or LRP-5 and confirmed that variants altering these structures result in decreased signaling activity via this essential pathway [39].

**Figure 6:**
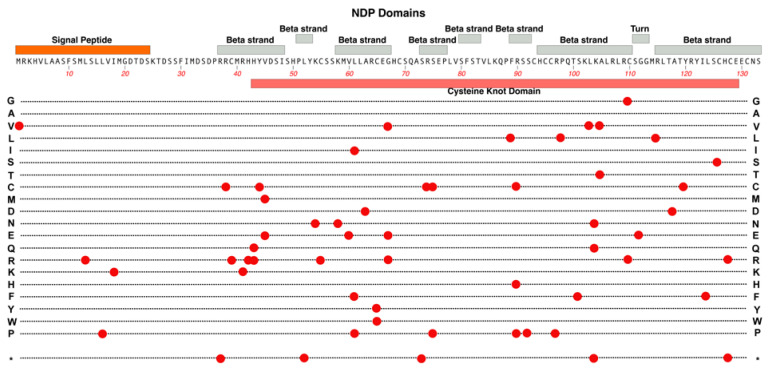
Structure and functional domain map of variants in NDP (Norrin). Key features include an N-terminal signal peptide required to target Norrin for extracellular transport. Four longer and four shorter beta-strand regions are layered in the Norrin monomer and the entire tertiary structure is stabilized by multiple disulfide bridges. The so-called cysteine knot domain involves most of the mature Norrin amino acid sequence. Over 50 pathogenic and likely pathogenic variants are indicated by red circles, marking the variant amino acid linked to Norrie disease or FEVR. The bottom row * indicates the location of nonsense (stop codon) variants. These and other non-pathogenic variants can be explored in the UniProt database: https://www.uniprot.org/uniprotkb/Q00604 (accessed on 27 October 2023).

Structurally, NDP consists of two main domains: an N-terminal signal peptide which is involved in facilitating the protein’s extracellular export and a highly conserved cysteine-knot motif, which makes up the bulk of the protein. See Figure 6.

Protein variants that alter the cysteine-knot confirmation are associated with more detrimental outcomes involving dysplasia or dysgenesis of the retina (i.e., Norrie disease), while variants that are located outside the motif are more commonly seen in patients with FEVR, in which the retina is present, albeit incompletely vascularized [11]. FEVR-associated genetic changes include R121Q, which has been identified by multiple studies across ethnically diverse populations, and S57X and K104Q, which have been present in cases of both FEVR and Norrie disease [41]. This highlights the interconnectedness of the mechanisms that underlie these two conditions.

This signaling pathway is specific to the retinal endothelial cell by virtue of the unique combination available of all four members of the ligand/receptor complex in the retinal endothelial cell: Norrin, FZD4, LRP5, and TSPAN12. Genetic loss of function experiments in mice indicate that while there is some overlap of Norrin and Wnt7a/Wnt7b signaling systems in the retina and brain, Norrin is more important for the iBRB and Wnt7a/7b has the larger role in development of the Blood-Brain-Barrier [42]. Subsequently, changes to the structure of any of these four proteins have been linked to similar retinal vascular pathology, such as FEVR or ROP [43]. Interestingly, while FEVR’s presentation can differ greatly across individuals, studies in mice have indicated that loss of function in Norrin, FZD-4, or LRP5 can often result in similar clinical phenotypes [43].

Ohlmann et al. (2005) were the first to demonstrate that ectopic expression of Norrin restored normal retinal angiogenesis that is lost in *Ndp* knockout mice [44]. Mice with ec-topic expression of Norrin in the lens were created using the lens-specific chicken Beta-B1-Crystallin gene’s proximal promoter to drive recombinant Norrin expression in the lens. These mice were crossed with *Ndp*^y/−^ mice to demonstrate that Norrin could act at significant distance to rescue Norrin Wnt-signaling and development of the neural retinal vasculature and promote regression of the tunica vasculosa lentis. Our research group has demonstrated that a single intra-vitreal injection of Norrin protein can accelerate vascular regrowth and reduce inner-retina neuronal cell loss in the mouse oxygen-induced reti-nopathy model [6,45]. More recently, Pauzuolyte et al. (2023) have shown that intravenous treatment of *Ndp* knockout mice with an adeno-associated viral vector 9 (AAV9) could rescue the failed development of the microvasculature in the neural retina and cochlea [46].

Because of Norrin’s central role in the development of the iBRB and its involvement in the pathogenesis of various retinopathies, recent research has been centered on Norrin’s capacity to combat the effects of VEGF-induced capillary leakage. Using a diabetic retinopathy mouse model, these studies indicate that exogenous Norrin can help to restore the retinal endothelial cell junctions via the canonical Norrin Wnt-signaling pathway [47]. 

### 2.5. FZD4

As has been highlighted in this paper, the Wnt-signaling pathway is responsible for the maintenance of the blood-retinal-barrier in the eye as well as for governing the process of retinal angiogenesis in the developing retina. The *FZD4* gene on Chr-11 encodes Frizzled Class-4 Receptor, a member of the Frizzled gene family, which are 7-transmembrane domain proteins. FZD-4 is the central cognate receptor for binding Norrin and is required for retinal angiogenesis and formation of the iBRB [48,49]. Norrin’s binds to FZD-4 and co-receptors LRP5 and TSPAN12, and subsequently, changes to the structure of any of these four proteins can cause FEVR [43]. Interestingly, while FEVR’s presentation can differ greatly across individuals, studies in mice have indicated that loss of function in Norrin, FZD-4, or LRP5 can often result in similar clinical phenotypes [43].

**Figure 7:**
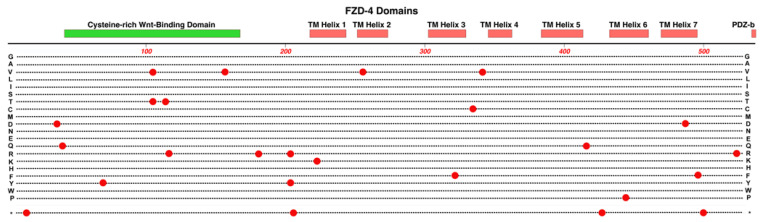
Structure and functional domain map of variants in FZD-4 (Frizzled-4). Key features include an N-terminal Wnt-binding domain, seven transmembrane helical regions, and a PDZ-binding domain. The locations of 25 pathogenic and likely pathogenic variants are indicated (red circles), showing the pathogenic amino acid variant. The bottom row * indicates the location of nonsense (stop codon) variants. These and other non-pathogenic variants can be explored in the UniProt database: https://www.uniprot.org/uniprotkb/Q9ULV1 (accessed on 27 October 2023).

*FZD4* has seven helix domains, a PDZ-binding domain, and a cysteine-rich Wnt-binding domain located near the N-terminus that is presented extracellularly and serves as the NDP binding site. See Figure 7. NDP is not known to bind to any other members of the Frizzled family of receptors, which highlights the specificity of the NDP/FZD-4 ligand/receptor relationship [43].

Some examples of single missense variations of *FZD4* that are linked to FEVR include M105V, R417Q, and G488D [50]. A sequencing survey of subjects with FEVR, Norrie disease, PFVS, Coats disease, and ROP found a strong statistical association of a double variant, p.[P33S(;)P168S], with ROP and a moderate statistical association with infant birth weight [51]. The locations of 25 pathogenic and likely pathogenic variants are shown in Figure 7. We expect that the sequencing of new FEVR subjects will continue to reveal novel pathogenic variants. One such recent example was reported by our group recently, Cys450Ter, a nonsense variant that generates an early termination codon within TM helix-6. This was predicted to result in the loss of TM helix-7 and the C-terminal PDZ-binding domain of the FZD4 protein [17].

### 2.6. LRP5

**Figure 8:**
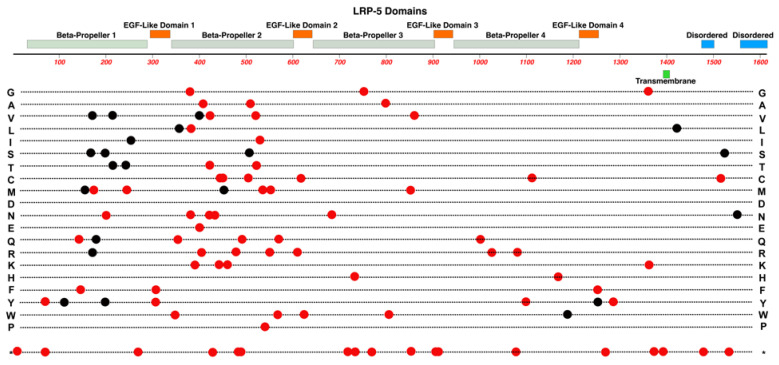
Structure and functional domain map of variants in LRP-5. Key features include the transmembrane domain, two intracellular disordered domains, and four extracellular Beta-Propeller/EGF-Like domain regions. Over 90 pathogenic and likely pathogenic amino acid variants are indicated, affecting the retina (red circles) and non-ocular syndromes (black circles, bone density). The bottom row * indicates the location of nonsense (stop codon) variants. These and many non-pathogenic variants can be explored in the UniProt database: https://www.uniprot.org/uniprotkb/O75197 (accessed on 27 October 2023).

The LDL-Receptor-Related Protein-5 is encoded by the *LRP5* gene on Chr-11 and is expressed in retinal endothelial cells. We refer the reader to He et al. (2004) for a more detailed review regarding the LRP-5 and LRP-6 receptors [52]. As noted above, in conjunction with TSPAN-12, LRP-5 acts as a co-receptor that enhances Norrin’s binding affinity for FZD4. LRP5 is required for vascular development in the deep plexus of the neural retina [53,54]. The LRP-5 protein has four extracellular Beta-Propeller/EGF-Like domain regions, one transmembrane domain region, and two intracellular disordered domains at the C-terminus. See Figure 8.

LRP-5 has direct interactions with Norrin (see Figure 5), using positively and negatively charged amino acid sidechains. The Beta-Propeller domains -1 and -2 nearer the extracellular N-terminus interact with the edge of the Norrin dimer, away from where FZD4 interacts with the central surface of the Norrin dimer. A ternary complex is formed once LRP-5 and FZD-4 are bound to Norrin [39]. Generally, the currently known distribution of pathogenic variants is greater in this region of the protein.

In addition to FEVR, pathologic mutations in LRP-5 are also associated with familial osteoporosis and high bone density syndromes [52]. Studies have identified several mutations in *LRP5* associated with FEVR. A few examples are L145F, R444C, A522T, T798A, and N1121D [55]. Qin et al. (2005) noted that these five missense mutations fall within the highly conserved Beta-Propeller domains of the LRP-5 protein [55].

### 2.7. TSPAN-12

**Figure 9:**
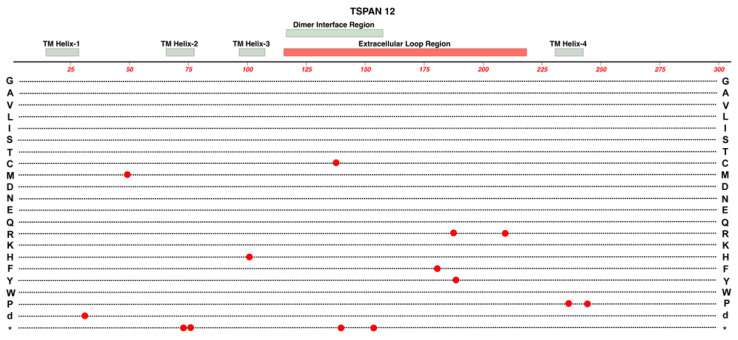
Structure and functional domain map of variants in TSPAN-12. Key features include the four transmembrane (TM) helical domains, the large extracellular loop region, and the dimer interface region. TSPAN-12 is a member of the Tetraspanin family of membrane proteins. Both the N-terminal and C-terminal domains are intracellular. The locations of 14 pathogenic and likely pathogenic variants are indicated by red circles with the variant amino acid indicated by the standard single-letter amino acid code. Row “d” indicates the location of a multiple amino acid deletion. The bottom row * indicates the location of nonsense (stop codon) variants. Exploration of additional non-pathogenic variants can be found in the UniProt database: https://www.uniprot.org/uniprotkb/O95859 (accessed on 27 October 2023).

TSPAN-12 is a member of the Tetraspanin protein family, which are characterized by four transmembrane domains with both their C-terminus and N-terminus being in the cytoplasm. These 4-transmembrane domains are linked by one small extracellular loop, one small intracellular loop, and one large extracellular loop. Interactions with other proteins and the variable roles of different TSPAN proteins are strongly impacted by amino acid sequence differences in the large extracellular loop [56]. See Figure 9.

*TSPAN12* variants can cause autosomal-dominant FEVR [57]. Variants of human TSPAN-12 have also been linked with ROP, specific types of cancer via regulation of progression, viral infections using TSPAN-12, and mental retardation [58,59,60]. Many variants identified in probands diagnosed with FEVR are predicted to result in a truncated protein, though studies were unable to perceive a difference in clinical presentation between patients with shortened proteins and those with missense mutations [57]. A237P is a variant observed in at least five families with FEVR [61].

Elegant work by Junge et al. (2009) used transgenic and knockout combinations in mice to demonstrate that normal vascularization of the neural retina required a full combination of Fzd-4, Lrp-5, and Tspan-12, as well as Norrin [62]. Loss of one allele of *Tspan-12* or *Lrp5* caused minimal reduction in vertical sprouts in the neural retina. Losing one allele each of *Tspan-12* and *Lrp5* had a much greater effect on this reduction. Complete lack of the Tspan12 protein in homozygous knockouts resulted in the failure to form vertical sprouts from the superficial plexus of the developing retinal vasculature. Furthermore, in cell-based transfection assays, TSPAN-12’s ability to enhance Norrin/beta-catenin signaling relied on the co-transfection of both FZD4 and LRP5.

Additional studies with *Tspan-12^(^***^−/−^**^)^ mice confirmed a significant increase in large retinal vessels and abnormal arterial-venous crossing along with upregulation of VE-Cadherin, which regulates iBRB integrity and EC inactivity [60]. These retinas also displayed a lack of intraretinal capillaries, and glomeruloid vessel malformations, which were like defects observed in pathogenic variants of *Ndp*, *Fzd4*, and *Lrp5*. Mural cells were also reduced or absent in some areas of the retinas of *Tspan-12^(^***^−/−^**^)^ mice. Additional features included microaneurysms, impaired hyaloid vessel regression, focal hemorrhages, retinal glial cell activation, and upregulation of plasmalemma vesicle-associated protein (PLVAP). The later effect was presumably due to impairment of Norrin signaling because Norrin Wnt-signaling is one factor thought to suppress PLVAP expression in retinal endothelial cells, which is essential to generate its high-barrier nature.

### 2.8. Notable Recent Genes Linked to FEVR Phenotypes

While this review has summarized the seven genes most associated with FEVR, above, current work continues to identify additional genes that may impact the neural retinal vasculature. Some recent notable genes include *EMC1,* which encodes the Endoplasmic Reticulum Membrane Protein Complex Subunit-1 protein, *CTNNA1*, which encodes α-Catenin, and *CTNND1,* which encodes the cell adhesion protein p120 (Catenin delta-1) [21,22,23].

### 2.9. EMC-1

Disease-causing variants in the *EMC-1* gene, on Chr-1, are mostly associated with impairment of neurological development and other multi-syndromic impacts on development. These include hypotonia, global developmental delay, scoliosis, and cerebellar atrophy [63]. This gene encodes the Endoplasmic Reticulum Membrane Complex (ERMC) Subunit-1 protein. The ERMC comprises ten subunits and this subunit is essential to insert the membrane domains of newly synthesized transmembrane proteins into the ER membrane. These would include single transmembrane domain proteins like LRP-5, tetraspanin proteins like TSPAN-12, and seven-transmembrane proteins like FZD-4, all required for Norrin Wnt-Signaling.

**Figure 10:**
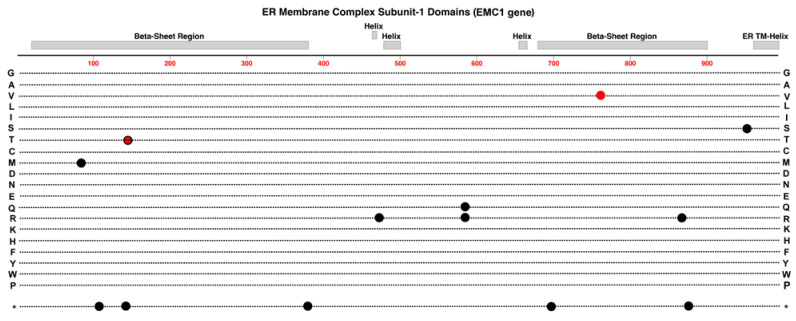
Structure and functional domain map of variants in Endoplasmic Reticulum Membrane Complex Protein Subunit-1. Key features include a C-terminal endoplasmic reticulum transmembrane helix and two large complex beta-sheet barrels joined by smaller helical domains. Pathogenic and likely pathogenic variants are indicated for those linked to FEVR (red circle), Retinitis Pigmentosa (red circle with black outline), and those linked to other developmental conditions (black circles). The bottom row * indicates the location of nonsense (stop codon) variants. Exploration of additional non-pathogenic variants can be found in the UniProt database: https://www.uniprot.org/uniprotkb/Q8N766 (accessed on 27 October 2023).

More recently, evidence from Li et al. (2023) suggests that specific variants of this gene can sometimes elicit a FEVR phenotype as well [23]. In mouse retinal endothelial cells, deletions in *EMC1* have been associated with abnormal retinal angiogenesis and the reduced proliferation of superficial vessels [23]. EMC1 protein was suggested to have a role in Norrin Wnt-signaling via upregulating the Norrin receptor, FZD4, via a post-transcriptional pathway. Whole exome sequencing (WES) of patients with FEVR identified a single novel missense variant in *EMC1*, I762V. When transfected into HEK 294T cells, the variant caused decreased protein levels of FZD4 [23]. This variant falls within the second large beta-sheet region of the subunit. (Figure 10) Moreover, the retinal phenotype observed in *EMC1* KO mice appears to be like that seen in mice with deficiencies in the any of the four main genes involved in the canonical Norrin Wnt-signaling (*NDP*, *FZD4*, *LRP5*, *TSPAN12*) [23]. Subsequently, the data from this study provide evidence for a novel candidate gene related to FEVR that deserves additional attention in future research.

### 2.10. CTNNA1

The α-Catenin protein is a Catenin family member quite different in structure from beta- and delta-Catenin. It plays a key role in mediation of Cadherin clustering coupled to actin cytoskeleton dynamics [64]. This is partly dependent on regions of the protein that interact with beta-Catenin and with alpha-Actinin. In effect, this protein provides at least one regulatory connection between Wnt-Signaling pathways and the assembly of E-Cadheren for adherens junction formation. Along with delta-Catenin, α-Catenin is another factor that associates with beta-Catenin and affects the available cytoplasmic pool of beta-Catenin for regulation of Wnt-signaling and regulation of adherens junctions.

**Figure 11:**
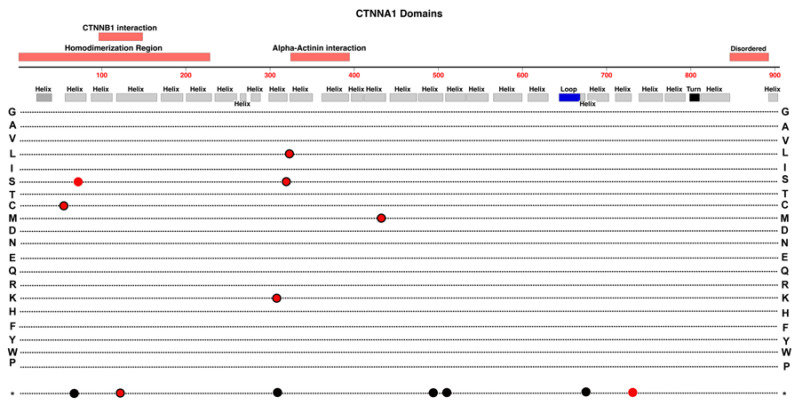
Structure and functional domain map of variants in CTNNA1 (Catenin alpha-1). Secondary structures are derived from Alphafold data (https://alphafold.ebi.ac.uk/entry/P35221 (accessed on 27 October 2023).) [68,69]. Pathogenic and likely pathogenic variants are indicated for those linked to FEVR (red circles), patterned macular dystrophy (red circles with black outlines), and those linked to non-retinal conditions including cancers (black circles). The bottom row * indicates the location of nonsense (stop codon) variants. Exploration of additional non-pathogenic variants can be found in the UniProt database: https://www.uniprot.org/uniprotkb/P35221 (accessed on 27 October 2023).

Previous research has linked α-Catenin, encoded by the *CTNNA1* gene on Chr-5, with a wide range of pathology, including gastric and breast cancers [65,66]. Moreover, butterfly-shaped pigment dystrophy has also been linked previously with variants in *CTNNA1*, suggesting that the gene may be involved in the development of retinal structure [67]. The tertiary structure of this protein is formed by the interactions of no less than 27 alpha-helical regions. The first six helical domains form a homodimerization region, while interaction with CTNNB1 requires helical regions 3 and 4. A region including helix 11 and 12 is responsible for interactions with alpha-actinin. See Figure 11.

A recent study by Zhu et al. (2021) observed that the conditional knockout of *CTNNA1* in mouse endothelial cells resulted in retinas that exhibited delayed peripheral vascularization and blood vessel leakage, thus partially mimicking the clinical phenotype observed in patients with FEVR [22]. Whole exome sequencing of patients with FEVR identified three novel variants in *CTNNA1* that appear to be associated with the disease [22]. Compound heterozygous mice were created by crossing *CTNNA1* conditional knock out mice with mice carrying one of the discovered variants, F72S, resulting in a phenotype that was like that observed in mice with homozygous conditional knockout of *CTNNA1* in retinal endothelial cells [22]. Specifically inhibited vascular development progress compared to wild-type mice. These mouse models displayed substantial amounts of avascular peripheral retina by age P7, compared to wild-type retinas where the superficial vasculature has normally reached the peripheral edge of the neural retina. The inhibition was substantial for heterozygous Ctnna^F72S/+^ mice but less severe than conditional heterozygous knockout Ctnna1^iECKO/+^ mice. A family with a heterozygous R376Cfs*27 variant had the most severe FEVR phenotype, congruent with the fact that this variant results in loss of functional α-Catenin. Wnt-reporter cell-transfection activation assays in HEK293 cells showed that the α-Catenin variants increased beta-Catenin-dependent reporter activation in contrast to wild type α-Catenin that reduced Wnt-reporter activity. Those results support the hypothesis that α-Catenin variants can impact retinal vascular development through a mechanism that alters normal beta-Catenin-mediated signaling [22].

### 2.11. CTNND1

**Figure 12:**
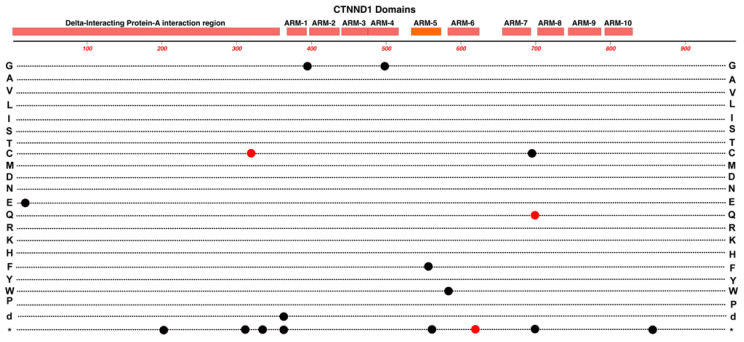
Structure and functional domain map of variants in CTNND1 (Catenin delta-1). Pathogenic and likely pathogenic variants are indicated for those linked to FEVR (red circles) and those linked to other developmental conditions (black circles). The bottom row * indicates the location of nonsense (stop codon) variants. Exploration of additional non-pathogenic variants can be found in the UniProt database: https://www.uniprot.org/uniprotkb/O60716 (accessed on 27 October 2023).

Variants in this gene are previously known for causing craniofacial, cardiac syndromes, and other developmental conditions [70]. The *CTNND1* gene (Chr-11) encodes for the Catenin delta-1 protein (p120-Catenin). This protein is another member of the catenin protein family and contains armadillo repeat domains that facilitate its interaction with other proteins including E-Cadherin (Figure 12). Association or displacement from E-Cadherin regulates the stability of E-Cadherin in adherens junctions [71]. The p120 protein has other interactions and roles that regulate epithelial and endothelial barriers. These include its selective inhibition of RhoA, which appears to be mutually exclusive of p120-Catenin interaction with E-Cadherin, and which facilitates the exchange of RhoA at cell-to-cell contacts [72]. Studies in *Xenopus laevis* have revealed another interaction of p120-Catenin with the Kaiso repressor. This interaction serves to decrease the Kaiso-mediated repression of several canonical Wnt target genes including *c-Myc*, *Cyclin-D1*, *c-Fos*, and *Siamois* [73]. This apparently works in parallel to support the beta-Catenin and TCF/LEF de-repression of Wnt target genes. 

A sample of 140 families associated with FEVR were evaluated with WES analysis, resulting in the identification of three candidate variants in *CTNND1* [21]. Transfection of these variants (R317C, K623*, R700Q) into HEK293T cells was associated with a subsequent decrease in Wnt-signaling activity, suggesting that these may possibly be pathogenic variants associated with FEVR [21]. Moreover, the knockout of *CTNND1* in mouse retinal endothelial cells resulted in a compromised blood-retinal-barrier and delayed regression of the hyaloid vessels—both of which are seen in FEVR [21].

## 3. Conclusions

We can draw several conclusions from this brief review regarding the mechanisms underlying FEVR and related phenotypes that impact the development and integrity of the neural retinal vasculature.

1. As we survey the protein domain maps that we have presented of currently known pathogenic and likely pathogenic variants, we can conclude that almost any functional subdomain of these proteins can be involved. This is not surprising because important protein-interaction functions or structural functions exist throughout their entire amino acid sequences. There are few, if any, non-essential regions in the ten proteins reviewed. This would suggest that there may be many novel variants awaiting discovery in the human population, especially for more recently linked genes such as CTNNA1;
2. FEVR can involve the disruption of any one of several different functions in endothelial cells, not just those related directly to the Norrin Wnt-signaling pathway. This is established by disease-causing variants in ZNF408, KIF11, and EMC1. However, what all these genes and their protein products have in common is that they are particularly important for critical retinal endothelial cell functions. These include correct membrane insertion of transmembrane proteins in the ER, regulation of adherens and tight junctions, cell growth, cell proliferation, migration of endothelial cells during formation of the retinal vasculature, and maturation of a high-barrier character endothelium. It is possible, and expected, that we may continue to discover novel FEVR-linked genes and good candidates would be any gene that is particularly enriched in retinal endothelial cells versus other endothelial cells. However, more broadly expressed genes with roles in any of the above noted functions may be good candidates as well. That may include nine other genes for subunits of the Endoplasmic Reticulum Membrane Complex;
3. The multiple allele knockout studies by Junge et al. (2009) in mice suggested the possibility that more severe FEVR-like phenotypes could result from combinations of two or more different alleles that have a minimal impact alone [66]. Our group recently surveyed a cohort of FEVR patients and immediate relatives to confirm that the incidence of protein-altering variants in two or three different FEVR-linked genes was substantially greater than in the general population [17]. Thus, it is possible that combined mild-alleles might result in more severe phenotypes, but we have not yet described clear examples of this in FEVR. To explore such possibilities for this relatively rare condition, it will be helpful to apply genetic testing methods that survey as many genes as possible and to form global research collaborations to investigate genotypes and phenotypes from varied populations.

Rare inherited retinal vascular diseases such as FEVR, Norrie disease, and Persistent Fetal Vascular Syndrome have historically been difficult to study due to their scarcity in the population, their complex presentations, and subsequently, their challenging diagnosis. Moreover, these conditions are sometimes multigenic and it is possible that the severity of phenotypes may be impacted by combinations of two or more protein-altering variants that may have no phenotype, or a mild phenotype, on their own. Clear evidence of such multigenic contributions is yet to be described and deserves the attention of future investigations. While this highlights the challenges of genetic testing for patients with rare retinal pathology, it is imperative that efforts continue to elucidate the mechanisms that govern these conditions. We would predict that not all FEVR-linked genes have been uncovered at this point in time and many other novel variants of known FEVR-linked genes will continue to be found.
